# Unique visible-light-assisted field emission of tetrapod-shaped ZnO/reduced graphene-oxide core/coating nanocomposites

**DOI:** 10.1038/srep38613

**Published:** 2016-12-12

**Authors:** Chaoxing Wu, Tae Whan Kim, Tailiang Guo, Fushan Li

**Affiliations:** 1 Department of Electronic and Computer Engineering, Hanyang University, Seoul 133-791, Korea; 2 Institute of Optoelectronic Display, Fuzhou University, Fuzhou 350002, People’s Republic of China

## Abstract

The electronic and the optoelectronic properties of graphene-based nanocomposites are controllable, making them promising for applications in diverse electronic devices. In this work, tetrapod-shaped zinc oxide (T-ZnO)/reduced graphene oxide (rGO) core/coating nanocomposites were synthesized by using a hydrothermal-assisted self-assemble method, and their optical, photoelectric, and field-emission properties were investigated. The ZnO, an ideal ultraviolet-light-sensitive semiconductor, was observed to have high sensitivity to visible light due to the rGO coating, and the mechanism of that sensitivity was investigated. We demonstrated for the first time that the field-emission properties of the T-ZnO/rGO core/coating nanocomposites could be dramatically enhanced under visible light by decreasing the turn-on field from 1.54 to 1.41 V/μm and by increasing the current density from 5 to 12 mA/cm^2^ at an electric field of 3.5 V/μm. The visible-light excitation induces an electron jump from oxygen vacancies on the surface of ZnO to the rGO layer, resulting in a decrease in the work function of the rGO and an increase in the emission current. Furthermore, a field-emission light-emitting diode with a self-enhanced effect was fabricated making full use of the photo-assisted field-emission process.

Field emission has been extensively studied for its importance in both fundamental research and high-power device applications, such as microwave power tubes, terahertz generators, and X-ray generators^1^,^2^,^3^. Among the several types of field emitters, low-dimensional nanostructures have been found to be potentially useful due to their large field enhancement. Especially, zinc oxide (ZnO) nanostructures are high-performance field-emission sources due to their high thermal and mechanical stability, high oxidation resistance in harsh environments, low work functions and easy preparation. We have demonstrated that ZnO nanostructures, including nanorods, tetrapod-shaped ZnO (T-ZnO), nanoparticles, and ZnO film^4^,^5^,^6^,^7^, exhibit excellent field-emission properties. However, their relatively low conductivity and low aspect ratio have limited their emission current density and have hindered their practical applications in efficient field-emission devices. Control of the surface’s geometrical morphology by sharpening the nanotapers or by using coatings with sharp-tipped nanoneedles and with amorphous C, CN_x_, and NiO films have been investigated in order to improve the field-emission current density^8^,^9^,^10^,^11^. The doping of ZnO with group III (Al, In, and Ga) or group IV (Sn and Pb) elements also offer an effective approach to improving the conductivity of ZnO and their field-emission characteristics^12^,^13^. Especially, photo-assisted field-emission techniques have attracted a great deal of attention because a high emission current density can be achieved by using such techniques^14^,^15^. Furthermore, illumination by some means other than an applied electric field may be a promising, efficient, real-time measure for controlling and adjusting the emission current density.

Generally, ultraviolet light must be used to generate electron-hole pairs due to the relatively wide band gap of ZnO. Compared to the adoption of ultraviolet light, the use of visible illumination to enhance the field-emission performance of ZnO is a more energy efficient and environmentally friendly technique that poses no harm to humans. However, the realization of visible-light-assisted field emission is difficult due to relatively low energy of visible-light photons. Research on visible-light-assisted field emission has yet to be initiated, making the investigation of field emitters with visible-light-assisted performance indispensable.

Fortunately, graphene and graphene-based nanocomposites have opened a pioneering field in materials science with the prospect of developing a wide range of nanocomposites due to its unique mechanical, chemical, electronic, and barrier properties, along with its high aspect ratio and flexibility^16^,^17^,^18^. We have demonstrated that the electronic properties of graphene-based nanocomposites are controllable, which makes them promising for applications in diverse electronic devices^19^,^20^,^21^. Especially, the field-emission properties of ZnO/graphene nanocomposites have attracted much attention due to the potential achievement of emissions with high current density from field-emission devices. Theoretically, density functional theory shows that the electronic structure of ZnO/graphene is significantly modified by applied electric fields, indicative of an enhancement of the field-emission properties^22^. Electrons have also been experimentally proven to be able to pass through the Schottky barriers formed at the ZnO/graphene heterojunction via a field-emission process^23^. Furthermore, the graphene sheets in field-emission prototype devices can act as a buffer layer between the ZnO and the bottom electrode. The ZnO nanorod array was hydrothermally grown on one side of flexible reduced graphene sheets with a turn-on field of 2.1 V/μm and an emitting current density of 470 mA/cm^2^ at 3 V/μm, which result from the significant lowering of the barrier resistance at the ZnO/graphene-aluminum heterojunction^24^. ZnO/reduced graphene nanocomposites, in which reduced graphene sheets modify the interface to improve the field-emission property of ZnO, have been prepared on silicon substrates, and the turn-on field of the ZnO nanostructure is decreased from 8.01 to 2.72 V/μm because of the formation of the ZnO/graphene nanocomposites^25^. The reduced graphene film can be used as a bottom electrode, as well as a substrate, in flexible field-emission devices due to its large flexibility and high conductivity, and to the low contact barrier between the reduced graphene film and the ZnO nanowires. Flexible field-emission devices based on transparent and flexible ZnO nanowires/reduced graphene nanocomposites have been observed to undergo convex, flat, and concave deformations with low turn-on fields of 2.0, 2.4, and 2.8 V/μm, respectively^26^. However, we should note that the graphene sheets in the reported nanocomposites mostly act as supports and buffers. We also point out that graphene sheets, because of their excellent flexibility, can play an important role in providing a unique monatomic shell on which to coat semiconductor nanomaterials^27^,^28^,^29^. The turn-on field of field-emission devices at 1 μA/cm^2^ utilizing graphene sheets grown on the surfaces of ZnO nanowires by using a plasma-enhanced chemical vapor deposition has been reduced from 2.5 to 1.3 V/μm due to the small radii and the additional high density of the emission sites in the graphene sheets^30^.

We have demonstrated that the field-emission properties of T-ZnO can be improved by coating it with graphene-oxide (GO) sheets, which enhance the mechanical connection between the T-ZnO and the bottom electrode^31^. In addition to a modification of the morphology, a graphene coating can change the electronic properties of ZnO quantum dots due to electronic transitions between the ZnO and the graphene^28^. As a result, a reasonable conclusion is that a graphene coating can control the field-emission properties of ZnO by modulating the energy band structure, as well as the surface topography, of the nanocomposites.

In particular, ZnO with a tetrapod shape is an optimal emitter choice for large-size field-emission devices fabricated by using the screen-printing method, as one of the crystal whiskers should always protrude from the T-ZnO layer at a large oblique angle due to the unique tetrapod geometry, which should enable a large field enhancement factor and a low turn-on field to be obtained. As a result, the complex post-processing method, which is widely used for carbon-nanotube- and graphene-based field-emission devices fabricated by using the screen-printing method, is unnecessary for the fabrication of T-ZnO-based field-emission devices^6^,^32^. Nevertheless, the fabrication of T-ZnO/graphene core/coating nanocomposites, the modulation of their photoelectric properties, and their possible visible-light-assisted field-emission performances have not yet been demonstrated.

In this work, we report that the ZnO material, an ideal ultraviolet-light-sensitive semiconductor^33^, can exhibit properties that are highly sensitive to visible light when coated with reduced graphene oxide (rGO); such properties have never before been observed. In this paper, we present a simple and facile technique for the synthesis of T-ZnO/rGO core/coating nanocomposites, and we discuss the results of our investigation into the mechanism behind the high visible-light sensitivity of the nanocomposites fabricated using that techniques. Especially, visible-light enhanced field-emission of the nanocomposites was observed for the first time.

## Results

### Morphological characteristics

**Figure 1:**
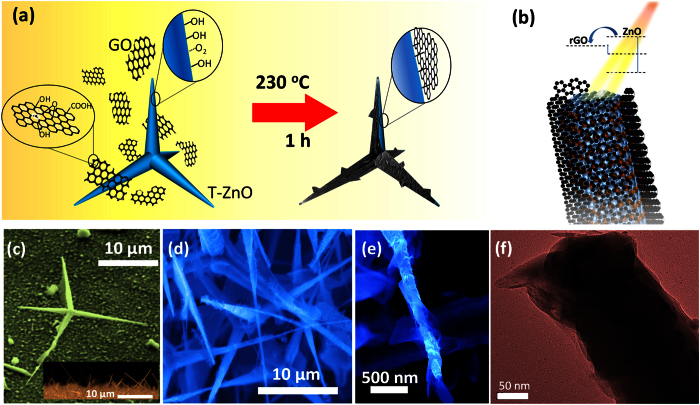
(**a**) Schematic of the process for synthesizing the T-ZnO/rGO core/coating nanocomposites by using a hydrothermal reaction. (**b**) Schematic of a ZnO crystal whiskers/rGO core/coating nanocomposite. (**c**) SEM image of the T-ZnO synthesized by using a vapor-phase method. The inset shows a cross-sectional SEM image of the T-ZnO layer. (**d**) SEM image of the T-ZnO/rGO core/coating nanocomposite. (**e**) High magnification SEM image of a single ZnO crystal whisker coated with rGO. (**f**) Transmission electron microscopy image of the T-ZnO/rGO core/coating nanocomposite.

T-ZnO was synthesized in a horizontal quartz tube furnace by using a vapor-phase method. The graphene oxide (GO) sheets were prepared from purified natural graphite by using a modified Hummers method (Supplementary Fig. S1). The hydrothermal-assisted self-assemble method was used for the formation of the T-ZnO/rGO core/coating nanocomposites, as shown in Fig. 1(a). The T-ZnO/rGO core/coating nanocomposites are schematically shown in Fig. 1(b). The as-synthesized T-ZnO products were loose, white, and cotton-like (Supplementary Figs S2 and S3). The detailed morphology of the T-ZnO was revealed by using scanning electron microscopy (SEM). The observed crystal whiskers had lengths in the range between 10 and 15 μm, as determined from the SEM image of a single T-ZnO layer in Fig. 1(c). The peculiar tetrapod structure of the T-ZnO may cause one of the crystal whiskers to protrude from the T-ZnO layer at a large oblique angle regardless of the random orientation of the T-ZnO, as shown the inset of Fig. 1(c) and Supplementary Fig. S4, which is beneficial for potential applications in field-emission devices. Because of their stereographic and loose configuration, the GO sheets in the solution were able to become in complete contact with the T-ZnO. A copper wire in a GO solution has been reported to be able to induce an aggregation of hydrothermally-reduced GO along the wire during a hydrothermal reaction process^34^, which is in reasonable agreement with our results. The role of the T-ZnO used in this work is the same as that of the copper wire, i.e, acting as a supporting template. Furthermore, the rGO most likely can connect to the ZnO via the chemical and the hydrogen bonds that have been generated from the Zn dangling bonds and the functional groups on the rGO^28^.

The geometrical morphologies of the T-ZnO/rGO core/coating nanocomposites can be modulated by controlling the concentration of the GO solution (Supplementary Figs S5–S8). The sample with the best field-emission properties was considered further because of its potential applications in field-emission devices (Supplementary Figs S9 and S10). Figure 1(d) shows an SEM image of a T-ZnO/rGO core/coating nanocomposite synthesized with a 4-mg/ml GO solution. The T-ZnO acts as a supporting structure and induces a connection of rGO sheets along it. The rGO sheets enwrap the surfaces of the T-ZnO crystal whiskers, as shown in the high-magnification SEM image in Fig. 1(e) and the transmission electron microscopy (TEM) image in Fig. 1(f); some sharp edges of the rGO sheets are seen to protrude from the T-ZnO crystal whiskers. As a result, the T-ZnO/rGO core/coating nanocomposites can act as excellent field emitters due to their large electric enhancement factor and large number of emission sites, which will be discussed later.

### Compositional and photoluminescence analysis

**Figure 2:**
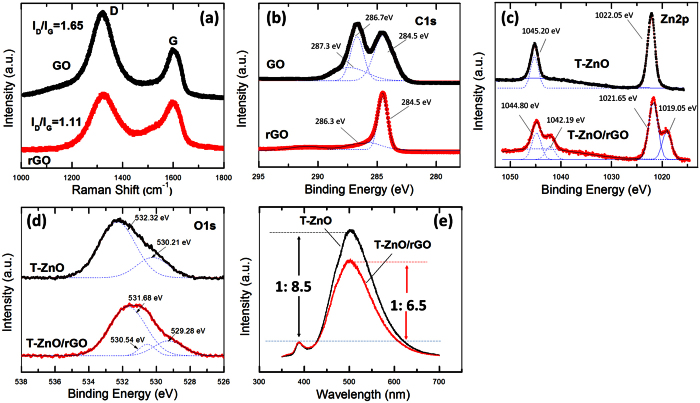
(**a**) Raman spectra of GO and rGO. (**b**) C 1 s binding energy spectra of GO and rGO. (**c**) Zn 2p and (**d**) O 1 s binding energy spectra of pure T-ZnO and T-ZnO/rGO core/coating nanocomposites. (**e**) Normalized PL emission spectra of pure T-ZnOs and T-ZnO/rGO core/coating nanocomposites.

During the hydrothermal reaction, the color of the GO solution changed from yellow brown to colorless with a little black powder (Supplementary Fig. S11). The black powder serves as evidence for a partial restoration of the conjugation network within the two-dimensional carbon structure. The rGO sheets in the solution were found to aggregate as black powders due to their low solubility in water under hydrothermal conditions. Raman spectroscopy was used to characterize the rGO, and the result is shown in Fig. 2(a). For comparison, the Raman spectrum of pure GO is also shown in Fig. 2(a). The typical features in the Raman spectrum for pure GO at 1596 cm^−1^ and at 1322 cm^−1^ are attributed to the G band and the D band, respectively. The prominent D band is related to the disorder in the graphene, which originates from defects associated with vacancies, grain boundaries, and amorphous carbon species^35^. The intensity ratio of the D band to the G band (I_D_/I_G_) of GO is about 1.69, which was decreased to 1.11 after hydrothermal treatment at 230 °C for 1 h, indicating that the hydrothermal reaction had recovered the aromatic structure by repairing defects. Furthermore, X-ray photoelectron spectroscopy (XPS) analyses showed the reduction of GO by using a hydrothermal reaction method (Fig. 2b). The binding energy of C1s for GO can be divided into three peaks, which corresponds to the functional groups of carbon sp2 around 284.5 eV, epoxy/hydroxyls around 286.7 eV, and carbonyl around 287.3 eV^36^. The components of the graphitic carbon and the oxidized carbon in the GO used in the experiment, as determined from the C1s spectra, were approximately 45 and 55%, respectively. After hydrothermal reduction, the components of the graphitic carbon and the oxidized carbon in the rGO were about 71 and 29%, respectively.

XPS measurements were carried out to confirm the chemical bonding states of the T-ZnO/rGO nanocomposites. Figure 2(c) presents high-resolution scanning information on the elemental Zn in pure T-ZnO and in T-ZnO/rGO nanocomposites. The binding energies of the Zn 2p_3/2_ and the Zn 2p_1/2_ peaks for pure T-ZnO are located at 1022.05 and 1045.20 eV, respectively, exhibiting an energy difference of 23.15 eV, which is in reasonable agreement with the standard XPS spectrum of ZnO^5^. The high-resolution XPS spectrum of Zn for the T-ZnO/rGO nanocomposites shows a doublet, corresponding to the Zn 2p_3/2_ and the Zn 2p_1/2_ orbitals, at 1022.05 eV and 1045.20 eV, respectively. After a multi-peak Gaussian fitting, in addition to the two typical peaks for elemental Zn, other peaks corresponding to the Zn 2p orbital were found at 1019.05 eV (Zn 2p_3/2_) and 1042.19 eV (Zn 2p_1/2_). This novel spectrum indicates that two chemical states of Zn exist in the T-ZnO/rGO nanocomposites. The typical peaks correspond to the Zn-O bond. The latter peaks with negative shifts show that the electron cloud attracts Zn^2+^ due to the presence of the rGO coating. We speculate that chemical bonds between ZnO and rGO are built during the hydrothermal reaction and that the Zn 2p binding energies of 1019.05 eV and 1042.19 eV correspond to a Zn-C bond. The high-resolution O 1 s XPS spectra are shown in Fig. 2(d). The O 1 s signal for pure T-ZnO can be fitted by using two nearly Gaussian components centered at 530.28 and 532.32 eV, respectively. The peak at 530.28 eV is attributed to oxidized metal ions (O−Zn^2+^). The binding energy component at 532.32 eV might correspond to the existence of a weakly-bound oxygen species on the surface of the T-ZnO^5^. The O 1 s signal for the T-ZnO/rGO nanocomposites can be fitted by using three nearly Gaussian components centered at 530.28, 531.54, and 532.98 eV, respectively. Because the electron affinity of the carbon atom is larger than that of the zinc atom and is smaller than that of the oxygen atom, the binding energy component centered at 531.54 eV might correspond to the existence an O-C bond. The above results confirm that the graphene in the T-ZnO/rGO nanocomposites has a strong chemical interaction with ZnO and that it does not act only as a physisorption coating.

The PL spectrum was measured at room temperature under an excitation wavelength of 325 nm to further investigate the interaction between rGO and ZnO, and the results are shown in Fig. 2(e). The emitted PL intensity recorded around 388.2 nm corresponds to the near band-edge emission^37^. The strong, broad emission peak at around 500 nm originates from the recombination of photo-excited holes with singly-ionized oxygen vacancies (deep-level emission)^38^. Notice that the intensity of the emission in the visible region for T-ZnO is relatively strong due to the high concentration of structural defects. The two spectra are normalized to ensure that the intensity of the near band-edge emission for the pure T-ZnO sample is the same as that for the T-ZnO/rGO nanocomposites, as shown in Fig. 2(e). The relative intensity ratio of the near band-edge emission to the deep-level emission is calculated for pure T-ZnO and for the T-ZnO/rGO nanocomposites. While the intensity ratio for the T-ZnO sample is 1:8.5, that for the T-ZnO/rGO nanocomposites is 1:6.5, indicative of a slight decrease in the deep-level emission intensity. The intensity of the deep-level PL for the T-ZnO/rGO nanocomposites was quenched, and similar phenomena have previously been observed for ZnO/carbon nanotubes and ZnO/rGO hybrids^39^,^40^. During excitation, more excited electrons are thought to be transferred from the ZnO to the rGO, which acts as an electron acceptor^40^,^41^.

### Sensitivity to visible light

**Figure 3:**
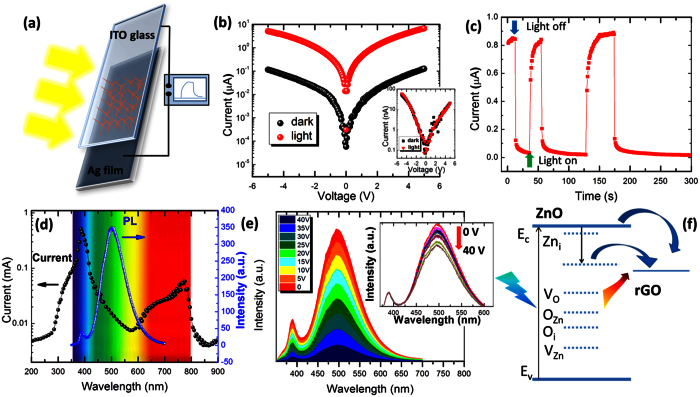
(**a**) Schematic configuration of a visible photoconductor based on T-ZnO/rGO core/coating nanocomposites. (**b**) *I–V* curves for T-ZnO/rGO core/coating nanocomposites in the dark and under 100-mW/cm^2^ (AM 1.5 G) irradiation. The inset shows the *I–V* curves for pure T-ZnO in the dark and under 100-mW/cm^2^ (AM 1.5 G) irradiation. (**c**) Pulse response of T-ZnO/rGO core/coating nanocomposites under 100-mW/cm^2^ irradiation. (**d**) Photoconductivity and PL spectrum of T-ZnO/rGO core/coating nanocomposites. (**e**) PL spectra of T-ZnO/rGO core/coating nanocomposites under various biases. The inset shows the normalized PL spectra. (**f**) Schematic band diagram of T-ZnO/rGO core/coating nanocomposites under illumination. The levels of the main native defects in ZnO are obtained from the literature^48^.

The detection of ultraviolet light by using ZnO nanomaterials has been extensively studied. However, few reports on the photo-response of ZnO to visible light can be found. Our study shows that T-ZnO/rGO core/coating nanostructures are highly sensitive to visible light. A solar simulator was used to test the response of the T-ZnO/rGO core/coating nanocomposites to visible light, and the results are shown in Fig. 3(a). Figure 3(b) shows the *I–V* curves of the T-ZnO/rGO core/coating nanocomposites with and without illumination. The conductance of the T-ZnO/rGO core/coating nanocomposites increased from 0.025 μS in the dark to 1.35 μS under 100-mW/cm^2^ (AM 1.5 G) irradiation at a 5-V bias, which indicated a high sensitivity to visible light. Time-resolved measurements of the photo-response to pulsed visible light were conducted, and the results are shown in Fig. 3(c). During the measurements, the indium-tin-oxide (ITO) electrode was at 2 V while the Ag electrode was at ground. This result shows that the conduction of the T-ZnO/rGO core/coating nanocomposites can be reversibly turned “on” and “off” by using a switching illumination.

The photoconductivity spectrum covering the range between 200 and 900 nm was measured in order to clarify the mechanism for the increased sensitivity of T-ZnO/rGO core/coating nanocomposites to visible light, and the results are shown in Fig. 3(d). The strongest conduction under 385-nm illumination appears to be due to the formation of photo-excited free electrons and to the desorption of oxygen^42^. We should note that even under lower-energy, that is, long-wavelength, illumination, the T-ZnO/rGO core/coating nanocomposites can support remarkable photocurrents. The T-ZnO/rGO core/coating nanocomposites under 775-nm illumination present strong conduction. The sensitivity to the red luminescence band, which cannot be observed in pure ZnO, is believed to result from an interaction with the rGO coating. Furthermore, the PL spectra of the T-ZnO/rGO core/coating nanocomposites under different bias voltages were measured, and the results are shown in Fig. 3(e). Interestingly, with increasing bias, the emission intensity clearly decreases. In particular, the broad emission peak at 500 nm very sensitively depends on the applied bias voltage, as shown in the normalized PL spectra in the inset of Fig. 3(e). That the surface adsorbed oxygen significantly affects the photo-response of ZnO nanomaterials is well accepted. Oxygen is chemisorbed on the surface of ZnO at vacancy sites, forming O^2−^ and resulting in a surface charge-depletion layer, thus leading to a reduction in the electrical conductivity. Photo-excited holes under illumination, such as ultraviolet light whose energy is sufficient to induce electron-hole pairs, discharge the adsorbed O^2−^ ions through surface electron-hole recombination while the photo-excited electrons significantly increase the conductivity. The electrons in the valence band under illumination by visible-light photons whose energies are below the Eg of ZnO cannot jump to the conduction band edge. Thus, pure ZnO nanomaterials cannot respond to visible light.

The rGO coating film in the T-ZnO/rGO core/coating nanocomposites acts as an electron acceptor, as shown in Fig. 3(f). Thus, the electrons in the oxygen vacancies on the surface of T-ZnO under visible illumination, which act as a donor level, can jump to rGO and leave holes in the surface of the T-ZnO. As a result, the surface holes discharge the adsorbed O^2−^ ions, leading to an increase in the conduction. Furthermore, the electrons in the shallow donor level can jump to the rGO, so the rGO induces a dissociation of the electron-hole exciton. Thus, the fluorescence, including visible emissions and near band-edge emissions, from T-ZnO/rGO core/coating nanocomposites is quenched. An increase in the applied electric field can also induce a transition of excited electrons from the ZnO to the rGO, resulting in a decrease in the PL density.

### Visible-light assisted field emission

**Figure 4:**
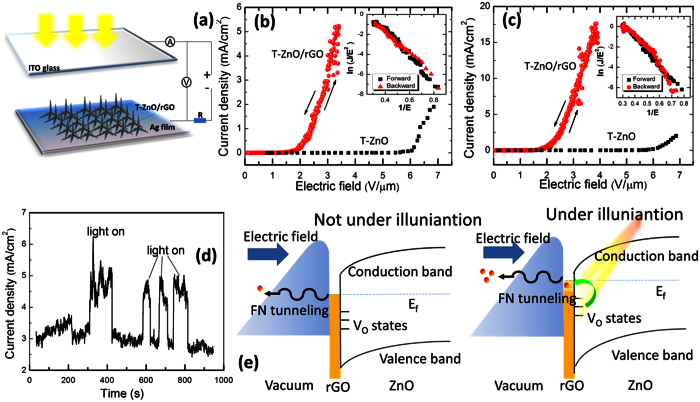
(**a**) Configuration for the visible-light enhanced field-emission measurements, in which the ITO glass is used as an anode. (**b**) Field-emission *J-E* curves for T-ZnO/rGO nanocomposites and pure T-ZnO in the dark. The inset presents related *F-N* plots for T-ZnO/rGO nanocomposites. (**c**) Field-emission *J-E* curves for T-ZnO/rGO nanocomposites and pure T-ZnO under visible illumination. The inset presents related *F-N* plots for T-ZnO/rGO nanocomposites. (**d**) Time-resolved field-emission response to pulsed visible light. (**e**) Schematic band diagram of T-ZnO/rGO nanocomposites in the dark and under visible illumination.

The T-ZnO/rGO core/coating nanocomposites can act as excellent electron field emitters due to their having a large electric enhancement factor and large number of emission sites. Furthermore, the effective photo-assisted electron transfer from the ZnO to the rGO can also improve the field-emission properties under visible illumination. A T-ZnO/rGO-based cathode was prepared to evaluate the field-emission properties of the T-ZnO/rGO core/coating nanocomposites, and their field-emission properties were measured by using a diode configuration, as shown in Fig. 4(a). The ITO glass used in this study acted as an anode, which allowed the T-ZnO/rGO nanocomposites to be exposed to light. The curves of current density versus applied electric field (*J-E*) for pure T-ZnO and T-ZnO/rGO nanocomposites with and without visible-light illumination are depicted in Fig. 4(b) and (c), respectively. The turn-on electric field (*E*_to_) is defined as the electric field required to obtain an emission current density of 10 μA/cm^2^. The threshold electric field (*E*_th_) is defined as the electric field corresponding to an emission current density of 10 mA/cm^2^. Obviously, illumination with visible light increased the emission current. The *E*_to_ for the sample in the dark was 1.54 V/μm while the *E*_to_ decreased to 1.41 V/μm for the sample under an illumination of 100 mW/cm^2^. With illumination, the current density was significantly increased from 5 to 12 mA/cm^2^ at an electric field of 3.5 V/μm. The *E*_th_ for the sample under illumination was 3.24 V/μm. That an emission current density of 10 mA/cm^2^ could not be obtained for the sample without illumination, even by increasing the applied electric field, due to the unstable emission under a high electric field is noteworthy. Of note also are the observations that the *E*_to_ for the pure T-ZnO sample in the dark was about 5.6 V/μm, and the field-emission performance of the pure T-ZnO sample was not enhanced under illumination with visible light.

The field-emission device was tested for its visible-light enhanced field-emission response, and the results are shown in Fig. 4(d). The field-emission current was recorded at an electric field of 3 V/μm. This result shows that the field-emission current of the T-ZnO/rGO core/coating nanocomposites can be reversibly turned “on” and “off” by using a switching illumination.

In general, cold field emission can be described by using the Fowler-Nordheim (*F-N*) equation in the following form^43^:


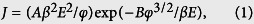


where *J* is the current density and E is the electric field strength. *A* and *B* are constants with values of 1.56 × 10^−10^ A∙V^−2^∙eV and 6.83 × 10^3^ V∙eV^−3/2^ μm^−1^, respectively. *φ* and *β* are the work function and the field enhancement factor of the emitters, respectively. This equation can be rearranged in *F-N* coordinates where the work function of the material is defined by the slope of the ln(*J*/*E*^2^) curve as a function of the reciprocal electric field *E*^−1^:


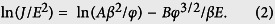


The insets of Fig. 4(b) and (c) show the corresponding *F-N* plots for the samples with and without illumination, respectively. The straight lines indicate the quantum-mechanical tunneling characteristics of the field emission. The field enhancement factor can be calculated by using the *F-N* equation. With *φ*_rGO_ = 5 eV^31^,^44^, the field enhancement factor of the sample in the dark is estimated to be about 6060. Note that *β* is related to the geometry, the crystal structure, and the nanostructure density of the emitters. The illumination did not change the cathode’s surface significantly, so the high field-emission current of the T-ZnO/rGO nanocomposites should be the result of a decrease in the work function. Based on the calculated field enhancement factor, the work function for the T-ZnO/rGO nanocomposites under an illumination of 100 mW/cm^2^ is estimated to be about 4.35 eV.

This phenomenon can be explained on the basis of the energy-band diagram and basic electric field theory, as shown in Fig. 4(e). More electrons move toward the tips of the rGO with increasing electric field between the T-ZnO/rGO and the vacuum, and the strong electric field bends the surface barrier. When the T-ZnO/rGO nanocomposites are illuminated with visible light, a larger number of electrons in the oxygen vacancies on the surface of T-ZnO, which act as a donor level, can jump to the rGO. Therefore, more energy levels above the Fermi level are occupied, resulting in a decrease in the work function. As a result, more electrons can emit to vacuum under the same electric field.

**Figure 5:**
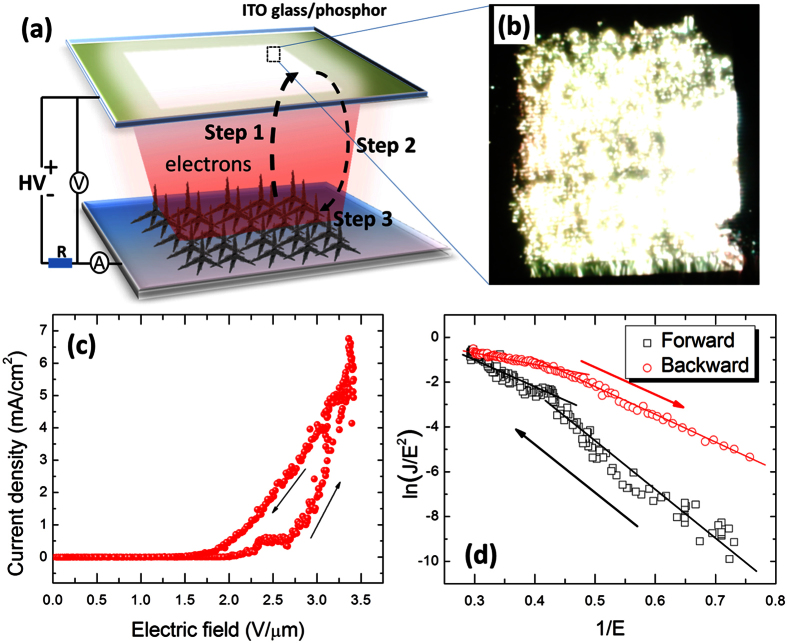
(**a**) Configuration of a field-emission light-emitting diode, in which phosphor-coated ITO glass is used as an anode. (**b**) Field-emission micrograph recorded from the phosphor layer. (**c**) Field-emission current density versus electric field with forward and reverse applied voltages. (**d**) *F-N* plots related to(**c**).

The emission performances of the T-ZnO/rGO nanocomposites can be improved by applying external illumination. Based on that principle, a positive-feedback process to self-increase the emission current can be obtained by depositing an electron-stimulated phosphor layer on the surface of the ITO, which is a field-emission light-emitting diode. Figure 5(a) shows a schematic of the structure of a field-emission light-emitting diode, in which the ITO anode is coated with an electron-stimulated phosphor layer. The electrons emitted from the T-ZnO/rGO nanocomposites might be accelerated in a strong external electric field (Step 1) and then hit the phosphor layer and induce visible luminescence (Step 2). The visible luminescence, in turn, enhances the field-emission current density of the light-emitting diode (Step 3). A bright luminescence (~300 cd/m^2^) is observed over the entire anode surface (Fig. 5(b)). Figure 5(c) presents the typical *J-E* curves under forward and reverse applied voltages. A prominent counterclockwise hysteresis is observed in the *J-E* curve. The related *F-N* plots are shown in Fig. 5(d). The difference in the slopes of the ln(*J/E*) versus 1/*E* curves for forward and backward applied voltages indicates changes in the characteristics of the work function and the field enhancement factor *β*. On the assumption that *β* remains constant, while the work function decreases with increasing forward applied voltage, work function increases with decreasing reverse applied voltage.

The origin of the field emission hysteresis behavior has been speculated to be due to several different factors, such as emission via intermediate electron energy states in the nanocarbon films, electrostatic alignment of the terminating species, capture of carriers by deep levels for wide-band-gap semiconductors, and adsorption/desorption of gaseous molecules^45^,^46^,^47^. However, the *J-E* curve of the T-ZnO/rGO nanostructure-based field-emission device with ITO glass as an anode shows no hysteresis (Fig. 5(b) and (c)). Therefore, the effect of luminescence from the anode’s surface might be dominant in the prominent counterclockwise hysteresis. More and more electrons are emitted from the graphene to vacuum with increasing forward applied electric field and hit the anode phosphor layer, leading to sufficient luminescence to, consequently, increase field emission. In the backward sweep, because the emission currents under reverse applied voltages are larger than those under forward applied voltages due to the luminescence from the anode, the hysteresis behavior of the field emission appears.

## Discussion

The tetrapod-shaped ZnO/rGO core/coating nanocomposites were fabricated by using a hydrothermal-assisted self-assemble method. The rGO shell in the nanocomposites has a strong interaction with ZnO, and it does not act only as a physisorption coating. Because of the strong interaction between the rGO shell and the ZnO core, a high sensitivity to visible light is obtained, which is not observed for pure ZnO. The high sensitivity to visible light results from photon-excited electrons jumping from oxygen vacancies on the surface of ZnO to the rGO. Furthermore, due to the electron transfer from ZnO to rGO under visible-light illumination, more energy levels above the Fermi level are occupied by electrons, leading to a decrease in the work function of graphene and a stronger field emission. As a result, we, for the first time, observed the field-emission properties of T-ZnO/rGO core/coating nanocomposites with the assistance of visible illumination. Overall, the T-ZnO/rGO core/coating nanocomposites and their visible-light-assisted field emission represent a more energy efficient and environmentally friendly approach, which poses no threat to humans, to enhancing the field-emission current density. These results also indicate that the photoelectric properties of ZnO/rGO nanocomposites can be modulated by using a graphene coating in the form of a core-shell structure, which opens the door to exploring functional optoelectronic materials.

## Methods

### Synthesis of T-ZnO/rGO core/coating nanocomposites

T-ZnOs were first transferred to the surfaces of the Ag electrodes by using a screen-printing technique. The screen-printing paste was a mixture of T-ZnO powder (200 mg), ethyl cellulose (90 mg), and terpineol (3.5 g). Then, the T-ZnO coated on the plate was baked at 400 °C for 30 min to remove the organic binders. After the GO suspension had been filled in a Teflon-lined autoclave reactor, the as-prepared T-ZnO-coated plate was intercalated. The reactor was sealed and was heated at 230 °C for 1 h. After the reaction had been completed, the plate was taken out from the reactor, washed with deionized water, and dried under ambient conditions. As a result, the rGO sheets were coating the surface of a ZnO whisker.

### Characterization of T-ZnO/rGO core/coating nanocomposites

SEM (Hitachi, S-3000N), TEM (JEOL, JEM-2010), Raman spectroscopy (Renishaw, inVia), and fluorescence spectrophotometry (Hitachi F-4600) were used to study the morphology, structure, and optoelectronic properties of the T-ZnO/rGO nanocomposites, respectively. The chemical bonding states of the T-ZnO/rGO nanocomposites were analyzed by using XPS (Thermo Scientific, ESCALab250Xi electron spectrometer) with incident Al Kα radiation, and the binding energies were referenced to the carbon 1 s line at 284.8 eV. The visible-light-sensitive conducting properties of the T-ZnO/rGO nanocomposites were measured with a Keithley 4200 SCS unit under 100-mW/cm^2^ (AM 1.5 G) irradiation from a solar simulator (ABET, SUN2000).

### Field-emission measurement

Field-emission measurements were carried out by using a diode configuration in a vacuum chamber evacuated to a base pressure of 2 × 10^−4^ Pa at room temperature. The plate coated with the as-formed T-ZnO/rGO nanocomposites was used as the cathode. ITO glass and the phosphor-coated ITO glass, which were parallel to each other and separated from the cathode plate by using 500-μm-height spacers, acted as anodes. The emission currents were measured by using an Agilent electrometer (34401A).

## Additional Information

**How to cite this article**: Wu, C. *et al*. Unique visible-light-assisted field emission of tetrapod-shaped ZnO/reduced graphene-oxide core/coating nanocomposites. *Sci. Rep.* **6**, 38613; doi: 10.1038/srep38613 (2016).

**Publisher's note:** Springer Nature remains neutral with regard to jurisdictional claims in published maps and institutional affiliations.

## Supplementary Material

Supplementary Information
