# Supplementary material for: Unique visible-light-assisted field emission of tetrapod-shaped ZnO/reduced graphene-oxide core/coating nanocomposites
Source: Sci Rep. 2016 Dec 12;6:38613. doi: 10.1038/srep38613 (PMC5150524; doi:10.1038/srep38613)
Supplement: Supplementary Information [file srep38613-s1.doc]

**Supplementary Information**

Fabrication of tetrapod-shaped ZnO/reduced graphene oxide core/coating nanocomposites and investigation of their unique visible-light-assisted field emission

Chaoxing Wu^1,2^, Tae Whan Kim^*1^, Tailiang Guo^*2^, Fushan Li^2^

^1^ Department of Electronic and Computer Engineering, Hanyang University, Seoul 133-791, Korea

^2^ Institute of Optoelectronic Display, Fuzhou University, Fuzhou 350002, People’s Republic of China

**Corresponding Author**

*To whom correspondence should be addressed. E-mail: twk@hanyang.ac.kr;

E-mail: gtl_fzu@hotmail.com


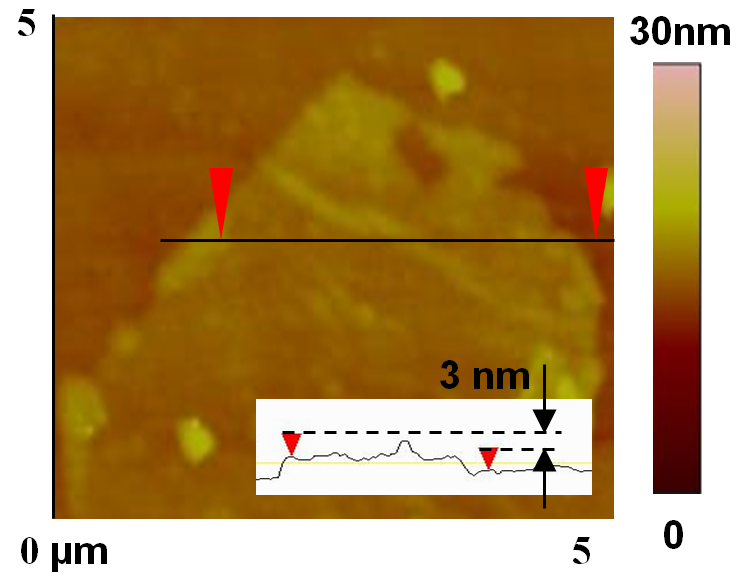


**Figure S1.** AFM image of GO sheet. The inset represents the cross-sectional analysis of the selected region between the two arrows. The thickness of the GO sheet is about 3 nm, as identified to be 2-3 layers.


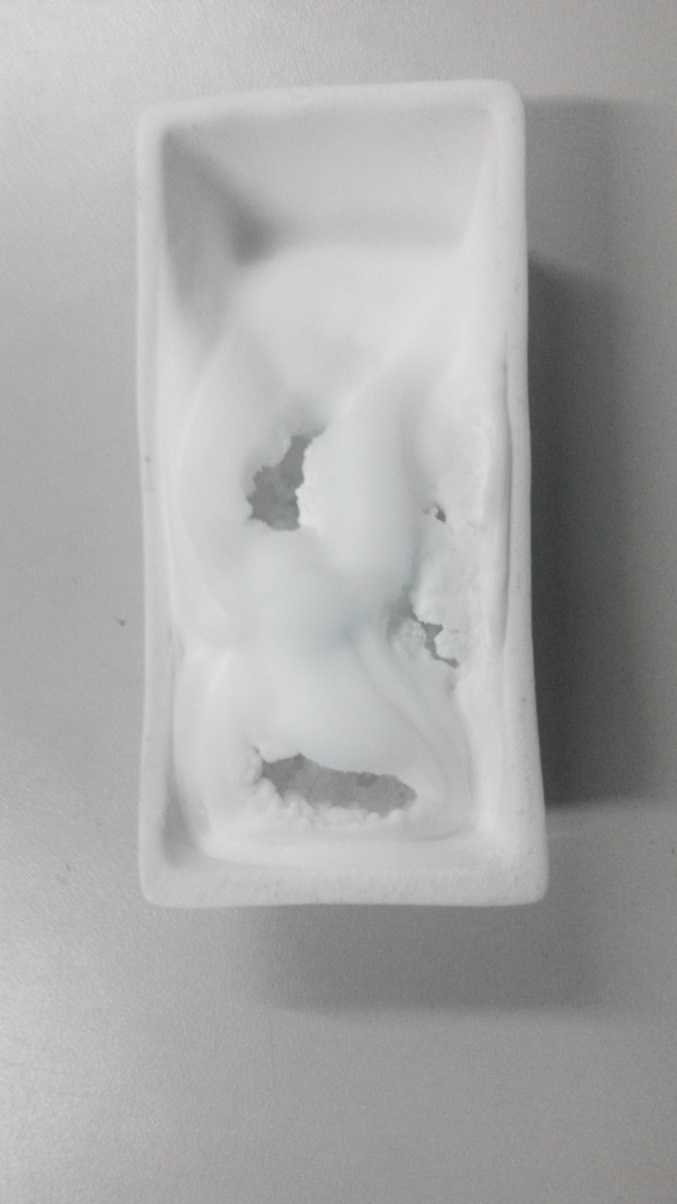


**Figure S2.** Photograph of the as-synthesised T-ZnO powder.


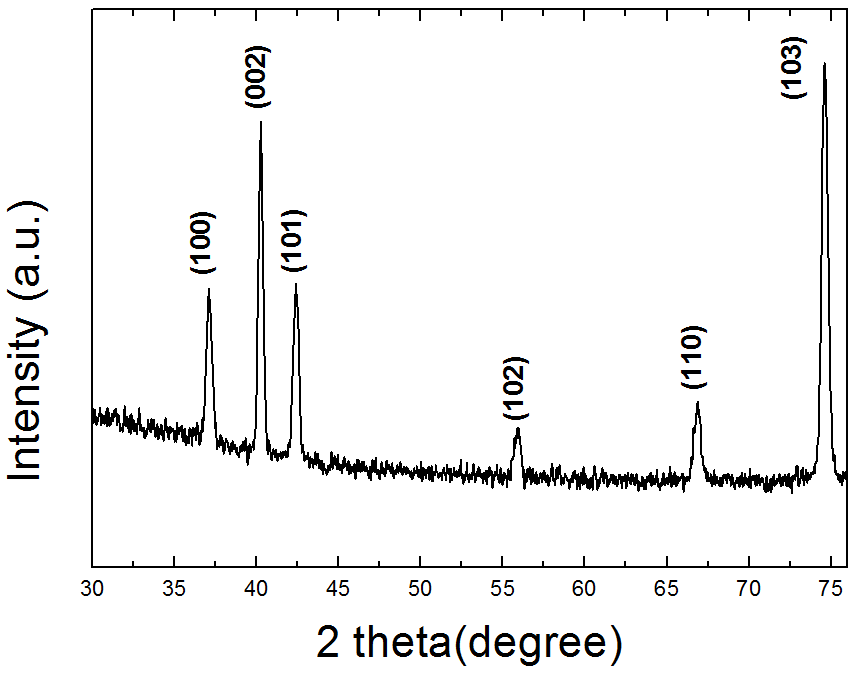


**Figure S3.** XRD spectrum of T-ZnOs. All dominant peaks can be referred to those of hexagonal wurtzite ZnO with lattice constants of a = 0.325 nm and c = 0.521 nm (JCPDS: 03-0888).


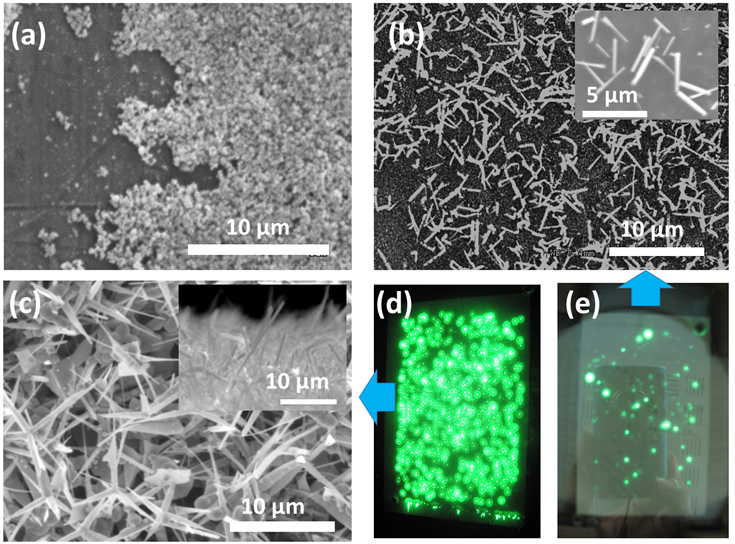


**Figure S4.** SEM images of (a) ZnO particles, (b) ZnO rods, and T-ZnOs transferred to the substrate by using a screen-printing method. (d) Field-emission pattern of T-ZnOs sample. (e) Field-emission pattern of ZnO rods sample.


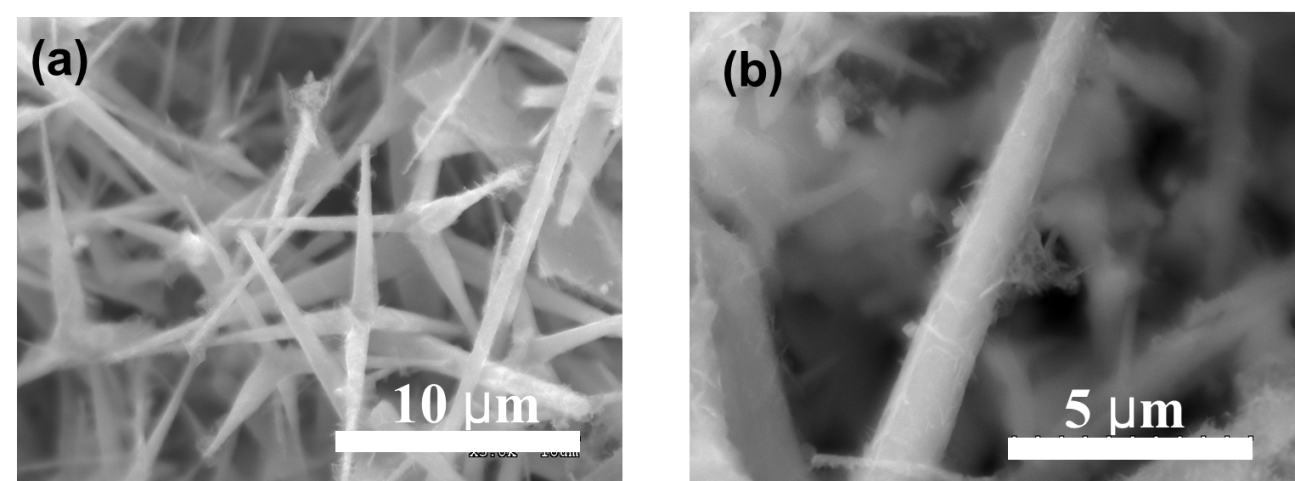


**Figure S5.** (a) Low- and (b) high-magnification SEM images of T-ZnO/rGO core/coating nanocomposites synthesized with 2 mg/mL GO solution. The surface of ZnO crystal whiskers are sparsely decorated with rGO sheets.


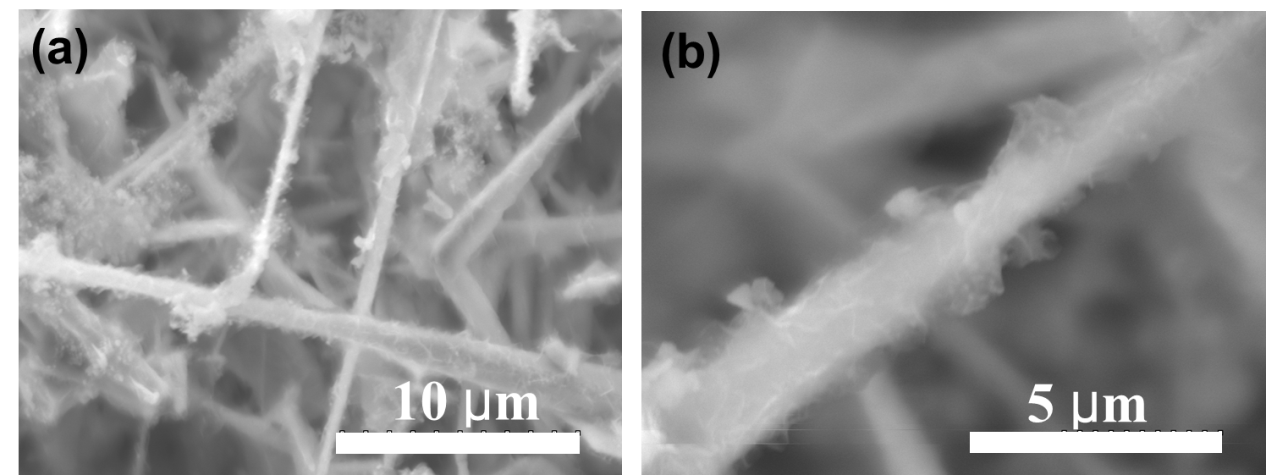


**Figure S6.** (a) Low- and (b) high-magnification SEM images of T-ZnO/rGO core/coating nanocomposites synthesized with 4 mg/mL GO solution. The surface of ZnO crystal whiskers are densely coated with rGO sheets.


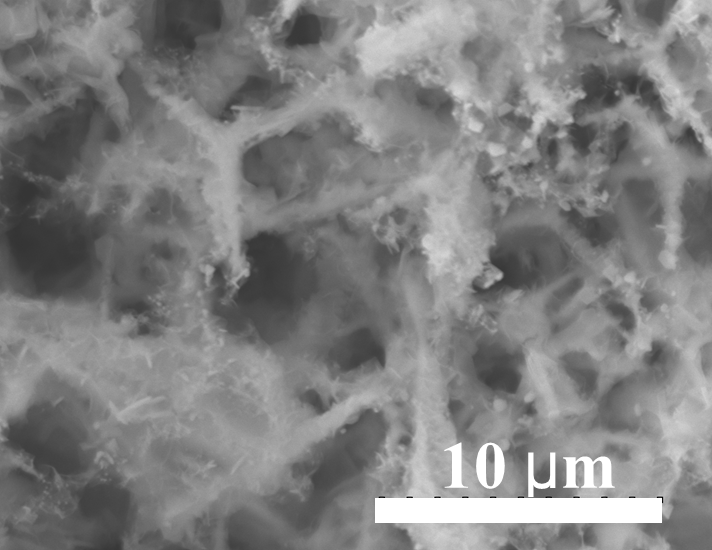


**Figure S7.** SEM image of T-ZnO/rGO core/coating nanocomposites synthesized with 6 mg/mL GO solution. The T-ZnOs are completely coated with fluffy rGO sheets.


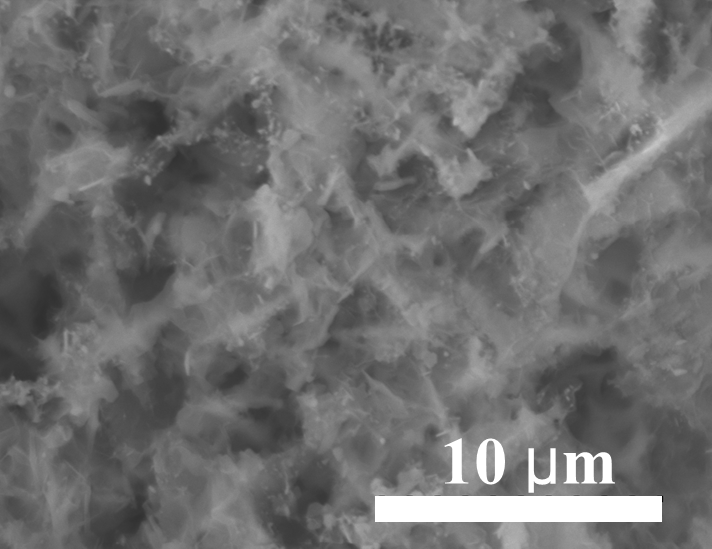


**Figure S8. S**EM image of T-ZnO/rGO core/coating nanocomposites synthesized with 10 mg/mL GO solution. The T-ZnO crystal whiskers are almost embedded in the rGO layer.


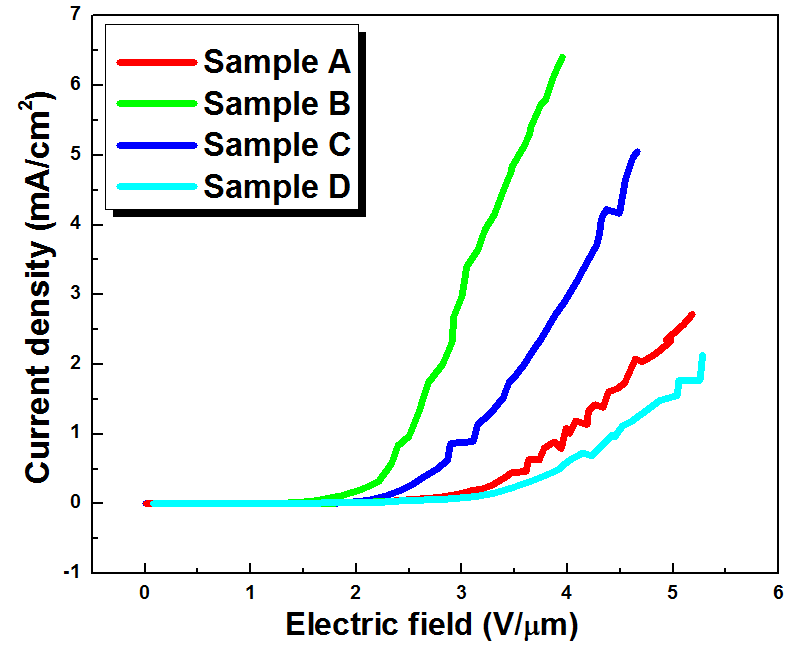


**Figure S9.** Field-emission properties of T-ZnO/rGO core/coating nanocomposites synthesized with 2 mg/mL GO solution (sample A), 4 mg/mL GO solution (sample B), 6 mg/mL GO solution (sample C), and 10 mg/mL GO solution (sample D). The highest emission current density suggests that the sample C synthesized with 4 mg/ml GO solution is more favourable than other samples for obtaining the optimum emission performance.


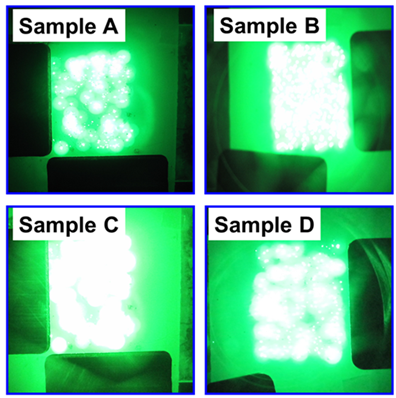


**Figure S10.** Field-emission patterns of T-ZnO/rGO core/coating nanocomposites synthesized with 2 mg/mL GO solution (sample A), 4 mg/mL GO solution (sample B), 6 mg/mL GO solution (sample C), and 10 mg/mL GO solution (sample D). All of the original emission current density was set as 1 mA/cm^2^. The sample C synthesized with 4 mg/mL GO solution was more favourable than other samples for achieving the optimum emission uniformity.


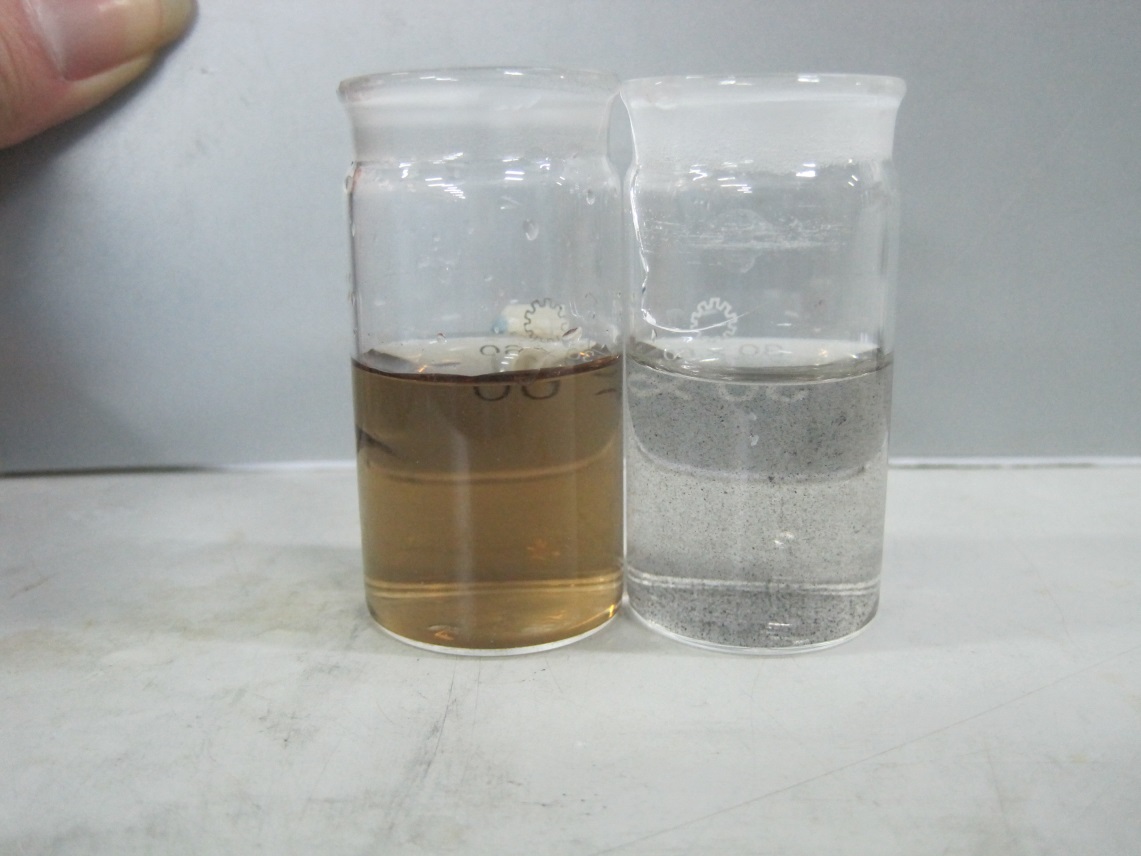


**Figure S11.** Photograph of the GO solution before (left) and after (right) hydrothermal reaction.
